# Quantitative Analysis of Tumor-Associated Mast Cells in Recurrent and Non-recurrent Urothelial Bladder Cancer in Stage pTa and pT1

**DOI:** 10.7759/cureus.14311

**Published:** 2021-04-05

**Authors:** Hristo Popov, Ina Kobakova, George S Stoyanov, Ekaterina Softova, Peter Ghenev

**Affiliations:** 1 General and Clinical Pathology, Forensic Medicine and Deontology, Medical University of Varna, Varna, BGR; 2 Pathology, Individual Medical Diagnostic Laboratory City Lab, Varna, BGR

**Keywords:** urothelial carcinoma, tumor-associated mast cells, local recurrence, statistical analysis, pathology

## Abstract

Background

Urothelial carcinoma of the urinary bladder (UCUB) is a common malignancy in both genders with a very high recurrence rate. There has been increasing evidence for a correlation between tumor-associated mast cells (TAMC) and tumor growth and recurrence rates. In the present study, we set out to establish a link between TAMC and the clinical morphological characteristics of UCUB in stages pTa and pT1.

Methodology

A retrospective non-clinical approach was used, with two groups of patients with UCUB. A total of 163 patients were included, 95 in the non-recurrent group and 68 in the recurrent UCUB group. Estimation of TAMC was performed on histological slides from the initial biopsy material using Giemsa and Toluidine blue staining. The collected data were statistically analyzed using the Kaplan-Meier curve, Mann-Whitney test, receiver operating characteristic curve, and chi-square analysis.

Results

Statistical analysis revealed that TAMC in the tumor stroma shows a positive correlation with local recurrence, with no statistical significance to the time of recurrence. No correlation showed statistical significance with pT stage, grade, gender, and age.

Conclusions

The amount of TAMC in UCUB correlates positively with the rate of local recurrence. The depicted correlations are similar to those established in mammary carcinoma, some lymphoproliferative disorders, and pancreatic and prostate malignancies.

## Introduction

Urothelial bladder cancer is relatively common and well known with a very high recurrence rate. Histologically, there are no objective criteria for assessing the tendency of local recurrence.

Apart from the cancer cell properties, such criteria might originate from the cells of the tumor microenvironment, and mast cells are considered as a promising candidate. There are numerous studies reporting that tumor-associated mast cells (TAMC) are concomitant with tumor growth [1,2]. A link has been established between the increased number of mast cells in the tumor and tumor aggressiveness [3]. In hematological malignancies, as well as in many solid tumors, the number of mast cells is strongly increased, which is mainly associated with disease progression. The mast cell count has been shown to correlate with the tumor stage, as well as with the prognosis and invasiveness of the tumor [4-7]. An increased number of mast cells were detected in Hodgkin’s lymphoma, large-cell B-cell non-Hodgkin’s lymphoma, primary skin lymphoma, pancreatic adenocarcinoma, prostate carcinoma, urothelial carcinoma, and many others [4-12]. An increased number of TAMC is interpreted either as an unsuccessful attempt of mast cells to counteract tumor growth or as an inhibition of their degranulation induced by the tumor [1,13]. Besides, TAMC in the stroma is contradictory as to whether they support or destroy tumor cells. In mammary cancers, TAMC promotes tumor development, invasion, and angiogenesis through locally increased growth factors such as stem cell factor and nerve growth factor. Because an extremely high recurrence rate is the most characteristic feature in the biological behavior of bladder urothelial cancer, we designed this study to evaluate quantitatively the presence of mast cells in the tumor stroma and their role in relapsing.

The present study aims to compare the clinical-morphological parameters of urothelial bladder carcinomas in stage pTa and pT1 with the number of TAMC in relapsing and relapse-free urothelial carcinomas.

## Materials and methods

Study design

A prospective, non-clinical approach was adopted for the study goals. The study was carried out on routine endoscopic biopsy materials at St. Marina University Hospital. All procedures carried out in the study fully adhered to the ethical standards of the Helsinki declaration of 1975 and its seventh revision from 2013. Medical University of Varna “Prof. Dr. Paraskev Stoyanov” Committee on Ethics approved the study (54/19.05.2016).

Patient selection

This study involved 163 patients with papillary bladder cancer in stage pTa and pT1 who were divided into two groups: The first group consisted of 95 patients diagnosed with bladder cancer from 2007 to 2011. These patients were followed for five years (2011 to 2016), and no histological or endoscopic evidence for relapse was present (control group). The second group included 68 patients diagnosed with bladder cancer for the same period (2007 to 2011), but with histologically verified local relapse.

All patients were monitored with cystoscopy, treated with transurethral resection, and subsequent intravesicular treatment with Bacillus Calmette-Guérin (BCG) vaccine. For both groups, the histological type, degree of differentiation, and tumor stage were determined. A comparative analysis of both groups was carried out on these indicators.

Histological slide preparation

The materials were fixed in 10% neutral buffered formalin for 18-24 hours, then embedded in paraffin blocks, following the standard procedure. Paraffin sections (4 µ thick) are stained routinely with hematoxylin and eosin (H&E). Visualization and counting of mast cells were performed through staining with Giemsa and Toluidine blue (pH 2.5) on the paraffin section.

Cell counting methods

All observations and mast cell counting were made using an Olympus BX50 light microscope (Olympus Optical Co, Ltd., Shinjuku, Tokyo, Japan). The slides were scanned with Leica Aperio ScanScope AT2 (Aperio Technologies, Vista, CA, USA) and visualized with ImageScope V12.1.0.5029 (Aperio) software.

The following indicators and criteria were evaluated on H&E-stained paraffin specimens:

Histological type of tumor. The diagnosis was made by applying the World Health Organization (WHO)/International Society of Urological Pathology (ISUP) classification for bladder tumors from 2004. Based on the Eighth American Joint Committee on Cancer 2018 it is defined as pT stage: pTa and pT1.

Degree of differentiation. The degree of differentiation of bladder cancer was determined according to the WHO/ISUP 2004 classification system separating low-grade tumors and high-grade tumors.

For evaluation of TAMC, mast cells in the peri and intratumor stroma were counted, and their density per square millimeter of tissue was calculated at magnification ×400.

Statistical analysis

The statistical analysis was carried out using the MS Excel 2016 programming product and the SPSS version 25 (IBM Corp., Armonk, NY, USA) statistical program for Windows. Descriptive analysis for determining statistical dimensions included mean [μ(X)], standard deviation (SD), minimum (min), and maximum value (max).

Cross-tabulation and chi-square test were performed for significant differences in the frequency performance of category values. Statistical significance in chi-square tests was considered at p ≤ 0. 05. Correlation analysis was conducted to assess the relationship between the indicators examined and to establish the strength of the interaction. The assessment of the strength of the relationship between variables was based on the results of Pearson’s r value. The degree of association between variables was defined as significant at r > 0.5 < r = 0.7, large at 0.7 < r = 0.9, and extremely large at r > 0.9 at p ≤ 0.05. Kaplan-Meier test was conducted for the survival of patients with urological carcinoma according to the influence of low and high expression of antibodies and the clinical-morphological indicators studied. Student’s t-test was conducted for comparing quantitative and qualitative indicators and examining the difference between them. The resulting data are presented as arithmetic mean and SD for the individual groups studied. Receiver operating characteristic (ROC) curve analysis was done to determine the role of accuracy and specificity of predictability of certain indicators. The value of the area under the curve was between 0.5 and 1.0. Complete separation of values by an indicator was done with a score above 0.75 or 75%.

In all analyses, a permissible level of significance of p < 0.05 at a 95% confidence interval (CI) was assumed. The graphical and tabular presentation of statistics was performed using Microsoft Office 2016.

## Results

A total of 163 patients with stage pTa and pT1 bladder cancer were included in this study. The average age of the patients was 65.92 ± 11.06 years, with the youngest being 31 and the oldest 89 years old. Patients were divided into six age interval groups, with the highest percentage in the 61-70-year group (63%) and the smallest in the 31-40-year group (1.8%). In the study group, out of 163 cases, 119 (73%) were men, while 44 (27%) were women.

Depending on the degree of invasion of the bladder wall in the study group patients in stage pTa and pT1 were selected, and they were subjected to intravesical local therapy and follow-up by cystoscopy. Urothelial carcinomas in the pTa stage were more common; of all the cases, 109 (66.9%) were in the pTa stage, while 54 (33.1%) were in the pT1 stage.

For papillary urothelial carcinomas in the pTa stage, we considered tumors in which no submucosal invasion was detected. For papillary urothelial carcinomas in stage pT1, we considered tumors in which submucosal invasion was detected.

The degree of differentiation of urothelial carcinoma of the bladder was determined by the 2004 WHO/ISUP classification. Accordingly, urothelial carcinomas in the study group were divided into two subgroups: low-grade and high-grade carcinomas. The majority of the patients were rated as low grade [107 (65.6%)], while patients with high-grade tumors were approximately twice less in number [56 (34.4%)].

The biological behavior of urothelial carcinoma of the bladder in stage pTa and pT1 is marked by more frequent local recurrence and less often invasion in the detrusor of the bladder wall. Therefore, in the present study, we divided patients into two groups with invasive and non-invasive tumors, and according to their biological behavior, into recurrent and non-recurrent tumors.

pT stage in patients with recurrent and non-recurrent urothelial carcinoma

Depending on the invasion of the bladder wall in the group of patients with tumor recurrence, 43 (63.2%) of the patients had a tumor defined as pTa and 25 (36.8%) patients had a tumor defined as pT1. In the group of patients without recurrence, 66 (69.5%) patients were in stage pTa and 29 (30.5%) patients were in stage pT1.

Regarding the recurrence in the study group using the Kaplan-Meier test to assess the prognostic value of the tumor stage (pT-stage), it was shown that invasive urothelial carcinomas (pT1) have a definite prognostic significance in the occurrence of local recurrence. There is a significant difference in the overall survival without local recurrence at different stages. The local recurrence in the group of patients with urothelial carcinoma in stage pT1 (8.12 months; 95% CI, 5.16-11.07) occurred much earlier than in patients with pTa stage (11.53 months; 95% CI, 8.03-15.03). Spearman’s statistical correlation analysis (rho) showed that there was no relationship between the stage of the tumor (stage) and the local recurrence (rho = 0.065; p = 0.407). The likelihood of local recurrence cannot be predicted according to the pT category.

Degree of differentiation (grade) in patients with recurrent and non-recurrent urothelial carcinoma

In the group of locally recurring cancer, the majority of patients were defined as low grade [38 patients (55.9%)] and high grade [30 patients (44.1%)]. In the group of carcinomas without local recurrence, most patients were defined as a low grade [69 (72.6%)] and approximately three times less were those with high-grade carcinoma [26 (27.4%)].

Regarding the time of relapse, the Kaplan-Meier test showed that low-grade urothelial carcinomas have a longer survival without local recurrence (11.44 months; 95% CI, 7.66-15.22) compared to high-grade urothelial carcinomas (8.8 months; 95% CI, 5.84-11.75).

Spearman’s (rho) statistical correlation analysis proved a connection between the differentiation (grade) and the local recurrence (rho = 0.174; p = 0.026.). Local recurrence of high-grade carcinoma was more likely to occur compared to low-grade carcinoma.

Mast cells

**Figure 1:**
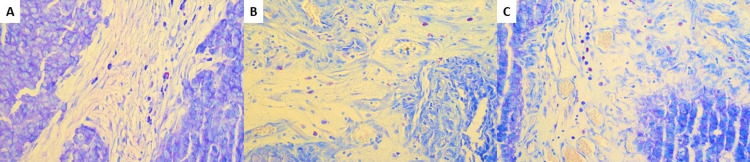
Mast cell distribution. High-grade urothelial carcinoma with an edematous stroma and sporadic mast cells, Giemsa stain, magnification ×400 (A). Low-grade urothelial carcinoma with an edematous stroma small groups of mast cells, Giemsa stain, magnification ×400 (B). High-grade urothelial carcinoma with an edematous stroma and mast cells forming trabecular structures, Giemsa stain, magnification ×400

Mast cells in the intra and peritumoral stroma are seen either as single cells (Figure 1A), diffusely scattered (Figure 1B), or form small groups (Figure 1C). Quantitative measurements of TAMC vary in urothelial carcinomas with and without recurrence. Table 1 shows that in urothelial carcinomas without recurrence the number of mast cells varies from 0 to 9.00/mm^2^.

**Table 1:** Number of mast cells in the intra- and peritumoral stroma in urothelial carcinomas without recurrence (n = 95).

	Patients	Minimum	Maximum	Mean	Std. Error
Mast cells/mm^2^	95	0.00	9.00	1.2547	2.08708

Table 2 shows the number of TAMC in recurrent urothelial carcinomas with varying numbers (0 to 30.70/mm^2^) and the months until recurrence. The earliest recurrence is one month after diagnosis, and the latest is 48 months after diagnosis. Quantitative analysis using the Mann-Whitney test showed that there was a significant difference in the number of TAMC in the stroma of primary tumors in patients with recurrent and non-recurrent pTa and pT1-stage carcinoma.

**Table 2:** Number of mast cells in the intra and peritumor stroma in urothelial carcinomas with recurrence and the months before its occurrence (n = 68).

	Patients	Minimum	Maximum	Mean	Std. Error
Mast cells/mm^2^	68	0.00	30.70	8.7647	6.48442
Time to local recurrence	68	1.00	48.00	11.8824	15.69916

**Figure 2:**
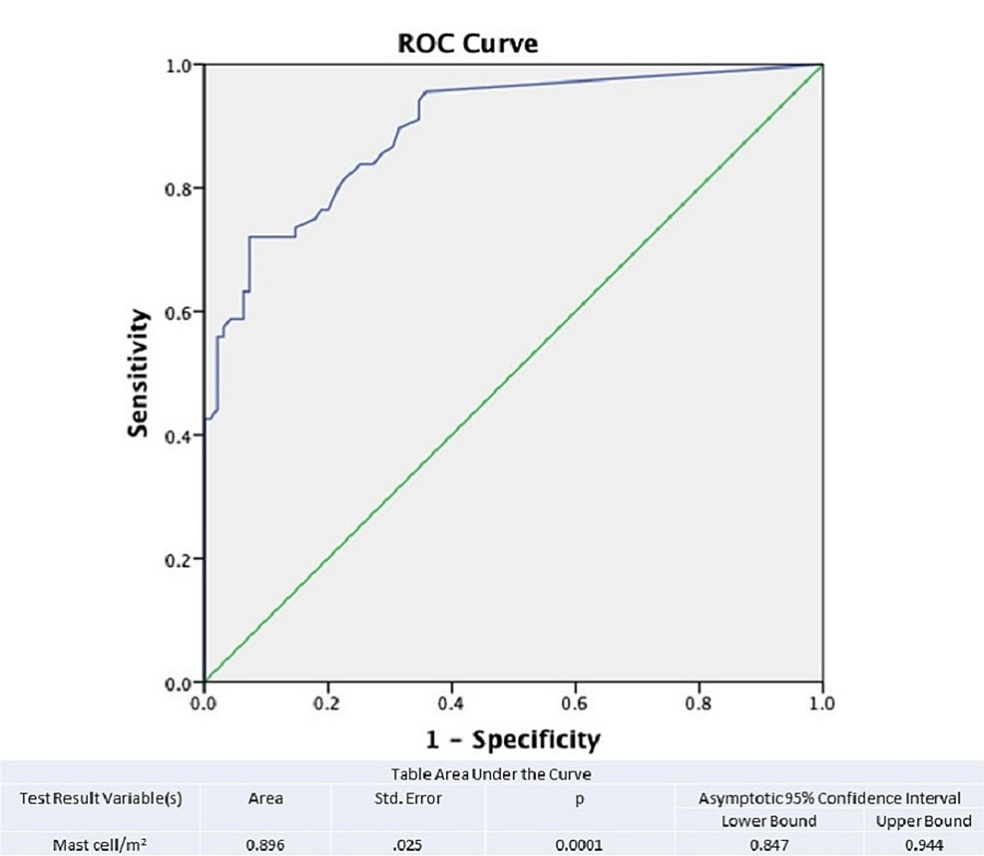
ROC curve analysis and determination of cut-off value for eosinophilic leukocyte count. ROC, receiver operating characteristic

To differentiate the patients and compare the results with the help of ROC curve analysis, we determined the cut-off value, which helped us to divide the number of mast cells in the tumor stroma into low and high. The established cut-off value of the number of mast cells was 4.38/mm^2^. The analysis showed that at optimal limit values ​​for the number of mast cells in the tumor stroma, the sensitivity was 75.4% and the specificity was 78.9% (AUC = 0.896; 95% CI = 0.847-0.944; p < 0.001) (Figure 2).

Quantitative assessment of TAMC and ROC curve analysis showed that there is a higher number of mast cells in the primary focus of urothelial carcinomas, which tend to recur at a statistically significant p value of <0.0001.

**Figure 3:**
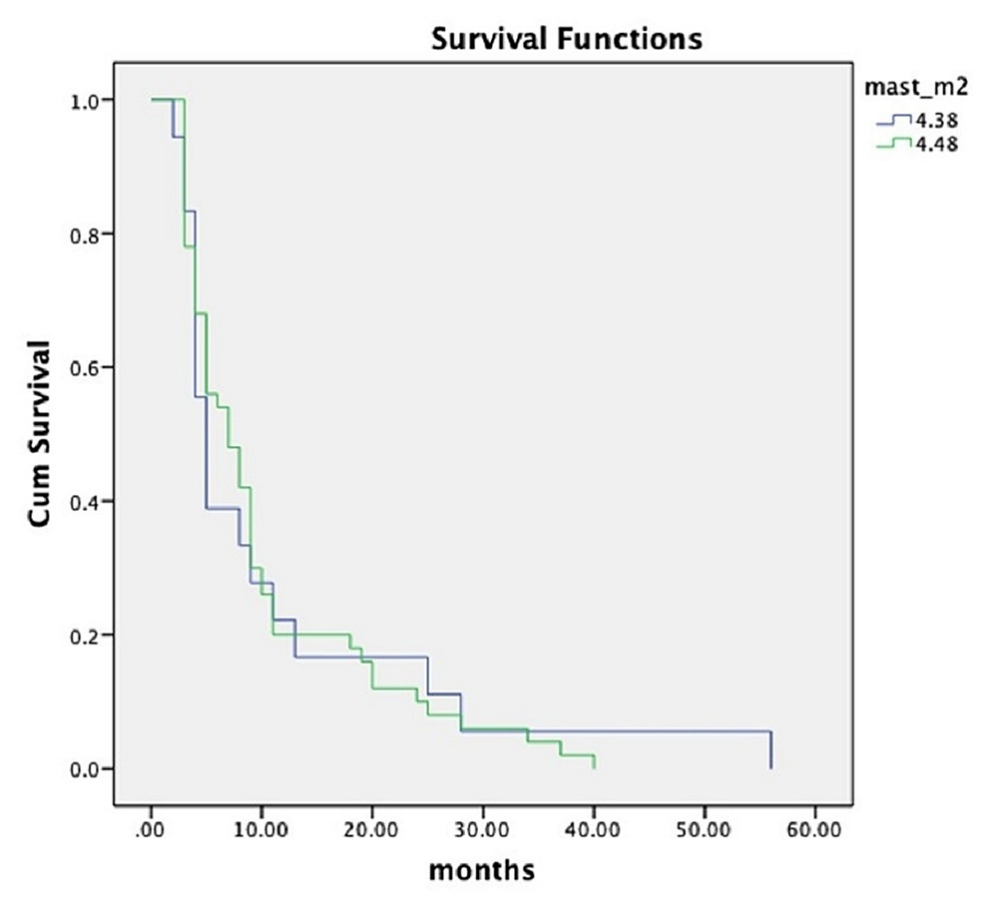
Kaplan-Meier curves of overall survival distribution of patients with and without recurrence of urothelial carcinoma according to mast cells in the tumor stroma.

The Kaplan-Meier curves show that patients with a high number of mast cells in the stroma did not have significant differences in survival without local recurrence compared to those with a low number of mast cells in the tumor stroma (Figure 3).

The ROC curve analysis shows that there was statistical significance (p < 0.0001) between the number of TAMC and the probability of recurrence. Kaplan-Meier curves show that there is no statistically significant difference between the number of TAMC and the months until local recurrence. Spearman’s rho test revealed a weak positive correlation between the increased number of mast cells and male gender (rho = 0.166; p = 0.034).

## Discussion

Our study included 163 patients, most of whom (n = 63) were in the age group 61-70 years, which coincides with data from GLOBOCAN, WHO, and the National Cancer Registry of Bulgaria. When comparing the groups of urothelial carcinomas with a recurrent and non-recurrent course, we found that, in recurrent urothelial carcinomas, the mean age is lower without statistically significant differences. We can assume that the occurrence of recurrent urothelial carcinomas at an earlier age is associated with faster disease progression. According to Shi, the duration of relapse-free survival decreases with age [14]. This is probably because bladder cancer tends to be well differentiated in patients under the age of 40 and the pTa stage in contrast to the older population. In our group, 14 (8.59%) patients under the age of 50 were initially diagnosed with non-invasive cancer, of which seven (4.29%) patients had a relapse (mean survival 13.6 months to recurrence). Patients younger than 50 years are usually in the pTa stage and low grade of differentiation compared to patients over 50 years of age. According to our study, the number of pT1 tumors that carry a higher risk of progression is significantly lower in younger patients.

Yossepowitch and Dalbagni reported that the higher incidence of high-grade tumors in older populations must be interpreted carefully as the tumor classification system has undergone several modifications over the years and there is variability in the degree of differentiation [15].

The age and sex of the patients do not influence the tendency to relapse. Our results show that the simultaneous comparison of the stage with the degree of differentiation in both groups shows high reliability, as more prone to recurrence are stage Ta urothelial carcinomas, which have a low degree of differentiation [16].

According to the results obtained, 121 (74.23%) patients developed urothelial carcinoma after the age of 60 and the male/female ratio was 3/1. These data coincide with the results of the literature [17,18]. The analysis of these data in the two groups shows that there is no statistically significant difference between them in terms of age and gender.

TAMC was present in both groups of patients with non-recurrent and recurrent urothelial carcinoma. Their absolute number and density were significantly lower in non-recurrent carcinomas. Both semi-quantitative and quantitative Mann-Whitney tests showed that there was a significant difference (χ^2^ = 13.49; p = 0.0001) in the number of TAMC in the stroma of primary tumors in patients from both groups. The same relationship was confirmed by ROC curve analysis. However, the number and density of mast cells are not related to survival without local recurrence. The Kaplan-Meier curves show that patients with high stromal mast cell counts did not have significant differences in survival without local recurrence compared to those with lower mast cell counts.

There is no satisfactory explanation for the increased number of mast cells in the stroma of recurrent carcinomas. The results presented here and the literature data are not enough to answer an important question, whether mast cells stimulate tumor recurrence, or are they an unsuccessful attempt to suppress the progression of the tumor?

Mast cells are highly reactive cells with multiple receptors and respond to a variety of stimuli influencing their maturation, activation, number, differentiation, biomolecular profile, and content [19,20]. Tryptase and chymase are the two main active substances studied, which have a proven protumorigenic effect. However, mast cells also possess other biomolecules that participate in tumor progression which are not well studied. This creates a prerequisite for more complete and in-depth future studies, mainly because targeted therapy is entering clinical practice, targeted precisely against this type of cell, which could favor prognosis in specific patients [21]. Histamine, excreted during mast cell degranulation, induces tumor cell proliferation through H1 receptors and inhibits the local effect of the immune system through the H2 receptors of tumor cells [1]. Mast cell mediators promote tumor spreading, particularly those localized in the central nervous system as they increase the permeability of the blood-brain barrier, especially in acute stressful conditions [1,13]. Perivascular mast cells in adenocarcinomas secrete several types of cytokines, proteolytic enzymes, and heparin, which to varying degrees can interfere with tumor development. Mast cell tryptase stimulates protease-activating receptors 1 and 2, which are also activated by thrombin and trypsin. Produced protamine binds exclusively to heparin thus neutralizing its anticoagulant properties, enhancing selective thrombosis in the vessels of the tumor, hypoxia, and cell death [22-26]. The contribution of mast cells to the development of tumors, whether positive or negative, needs to be evaluated regarding the tissue of origin of the neoplasm. Stimulated mast cells have antitumor potential through direct activation of cytotoxic lymphocytes [27]. On the other hand, data on their influence on angiogenesis are contradictory. It is believed that mast cells stimulate angiogenesis through several different mechanisms and it is even assumed that they trigger the “angiogenetic switch” [28,29]. Other authors suggest that under certain conditions and localizations TATM induces endothelial cell apoptosis [30].

## Conclusions

Although the suggested mechanisms are revealing different aspects of bladder cancer biology and need further clarification, the results presented here provide statistically significant evidence that TAMC in the primary biopsy is a reliable predictive factor for early relapse.
